# Risk factors for congenital heart disease: The Baby Hearts Study, a population-based case-control study

**DOI:** 10.1371/journal.pone.0227908

**Published:** 2020-02-24

**Authors:** Helen Dolk, Nichola McCullough, Sinead Callaghan, Frank Casey, Brian Craig, Joanne Given, Maria Loane, Briege M. Lagan, Brendan Bunting, Breidge Boyle, Tabib Dabir

**Affiliations:** 1 Institute of Nursing and Health Research, Ulster University, Newtownabbey, Northern Ireland, United Kingdom; 2 Department of Cardiology, The Royal Belfast Hospital for Sick Children, Belfast, Northern Ireland, United Kingdom; 3 School of Psychology, Ulster University, Coleraine, Northern Ireland, United Kingdom; 4 Department of Genetic Medicine, Belfast City Hospital, Belfast, Northern Ireland, United Kingdom; Technische Universitat München, GERMANY

## Abstract

We investigated the role of maternal environmental factors in the aetiology of congenital heart disease (CHD). A population-based case-control study (242 CHD cases, 966 controls) was conducted using an iPad questionnaire for mother with linkage to maternity and first trimester prescription records. Risk of CHD was associated with low maternal education (OR adjusted for confounders 1.59; 95% confidence interval [CI], 1.02–2.49), pregestational diabetes (OR 4.04; 95% CI 1.00–16.28), self-reported maternal clotting disorders (adjOR 8.55, 95%CI 1.51–48.44), prescriptions for the anticlotting medication enoxaparin (adjOR 3.22, 95%CI 1.01–10.22) and self-reported vaginal infections (adjOR 1.69, 95%CI 1.01–2.80). There was no strong support for the hypothesis that periconceptional folic acid supplements have a protective effect, but there was a protective effect of frequent consumption of folate rich fruits (adjOR 0.64, 95%CI 0.47–0.89). Compared to the most common pre-pregnancy dietary pattern, CHD risk was associated with a poor diet low in fruit and vegetables (adjOR 1.56, 95%CI 1.05–2.34). Mothers of cases reported more pregnancy related stress (adjOR 1.69; 95% CI 1.22–2.34) and multiple stressors (adjOR 1.94, 95%CI 0.83–4.53). We found no supportive evidence for CHD risk being associated with obesity, smoking, depression or antidepressant use in this population. Our findings add to the previous evidence base to show potential for public health approaches to help prevent CHD in future by modifying environmental factors. Independent confirmation should be sought regarding elevated CHD risk associated with maternal blood clotting disorders and their treatment, since we are the first to report this.

## Introduction

Significant progress has been made in recent decades in relation to the earlier detection of congenital heart disease (CHD), both prenatally and neonatally, while advances in surgical and other treatments have improved survival and quality of life. However, very little progress has been made in primary prevention–modifying risk factors to reduce the number of affected babies. The main exceptions are vaccination against congenital rubella [1]and the identification of maternal pregestational diabetes as a strong risk factor which can be managed by better glycaemic control in the periconceptional period [2].

Mechanisms for abnormal cardiac morphogenesis relate to disruption of the normal embryological process occurring within the first 8 weeks of gestation [3]. The cardiogenic cords arise from the mesoderm and develop a lumen prior to fusing to become the primitive heart tube at around 21 days of gestation. The heart tube undergoes alignment and septation between 24 and 35 days. Disturbances in alignment lead to major heart defects including various forms of Single Ventricle and Tetralogy of Fallot. Defects in septation may also occur at this stage while deficiencies in development of the cardiac inlet and outlet valves occur between 5 and 8 weeks of gestation. It is in early pregnancy therefore that we look at potential maternal environmental exposures which may disrupt cardiac morphogenesis.

The association of Down Syndrome and other aneuploidies with CHD is well known. The use of new genetic technologies has led to the identification of increasing numbers of babies with CHD who have copy number variations or point mutations [4]. Exclusion of genetic syndromes can potentially increase the sensitivity of aetiological research focusing on environmental (non-genetic) causal factors of interest, while it is recognised that most CHD is likely to be caused by multiple environmental and genetic factors acting together [4–6].

Scientific uncertainty surrounds the status of a number of common potentially modifiable environmental exposures as risk factors for CHD. These include the protective effect of periconceptional folic acid supplementation or fortification [7,8], maternal smoking [9,10], maternal obesity [11–14] and maternal depression or antidepressant use [15–19]. These are all major health determinants which negatively affect a range of pregnancy outcomes and can be tackled by public health programmes as well as individual healthcare.

In this paper we report the results of a population-based case-control study, the Northern Ireland Baby Hearts Study, which set out to test whether common risk factors (low folate/folic acid and its nutritional context, maternal smoking, maternal obesity, maternal antidepressant use and its mental health context, were associated with risk of CHD, as well as examining a range of other maternal diseases, medications and exposures as risk factors.

## Materials and methods

### Study design

We conducted a case-control study with hybrid data collection methods linking maternal iPad-assisted questionnaires (retrospective exposure data) to maternity and prescription records (prospective exposure data), as described in detail elsewhere [20].

### Case and Control definition and recruitment

Cases and controls were eligible if mothers were resident in Northern Ireland during pregnancy, aged at least 17 years and able to read English or Polish (the most common language among non-English speakers).

**Fig 1:**
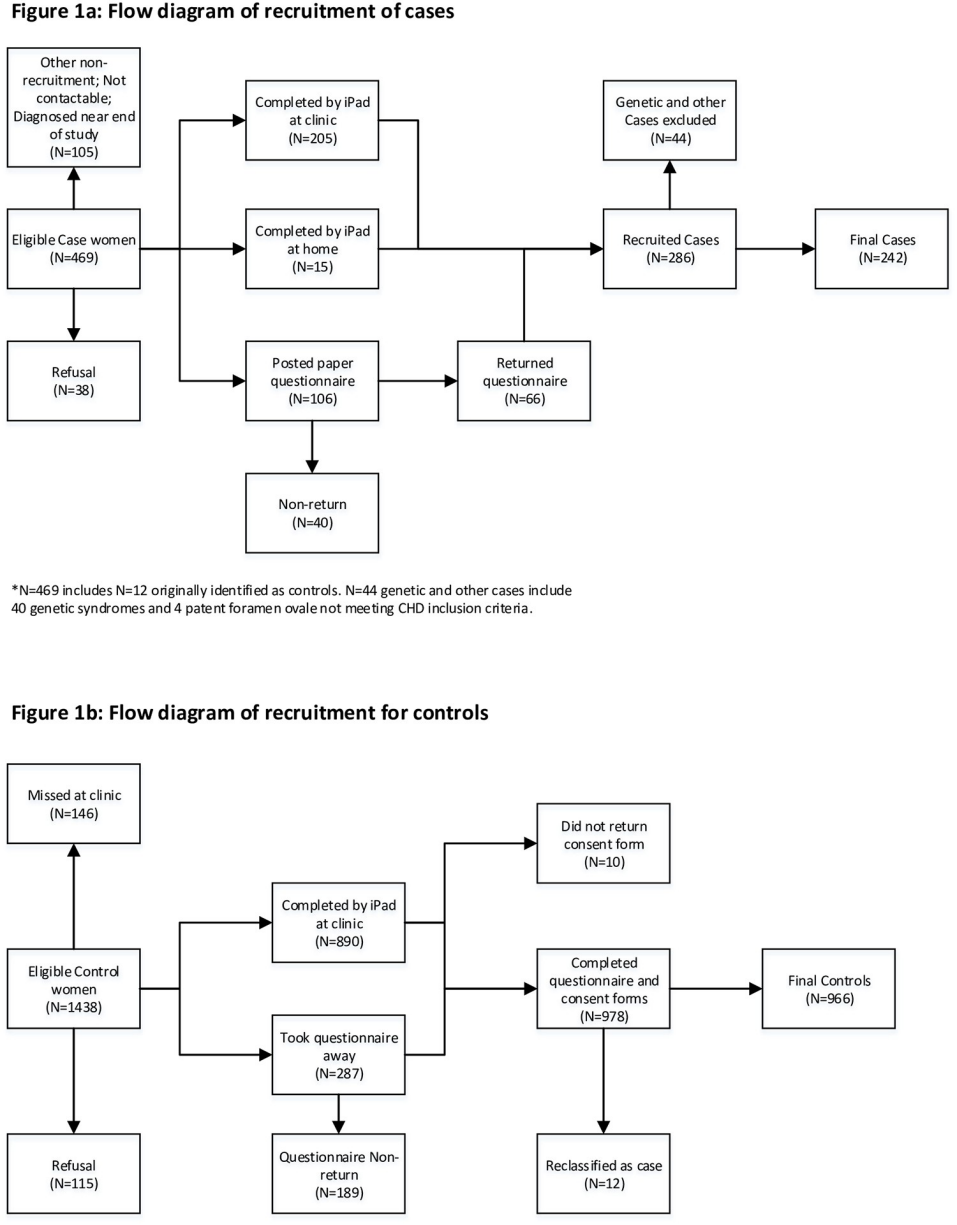
Flow diagram of recruitment for cases (a) and controls (b).

Cases were babies with a congenital heart defect diagnosed prenatally or before the baby was six months old in the single paediatric cardiology centre serving the entire Northern Ireland population. Babies with Trisomy 21, commonly associated with CHD, were excluded. Babies with other genetic syndromes diagnosed after referral to the clinical genetics service were also excluded (assessed by clinical geneticist TD on the basis of genetic and clinical information). Babies who were stillborn with CHD were eligible for inclusion if diagnosis had been made prenatally. Terminations of pregnancy for fetal anomalies are not legal in Northern Ireland. Cases of patent ductus arteriosus associated with preterm birth, patent foramen ovale or small Atrial septal Defect (ASD 4mms or less on 2-dimensional echocardiography) were excluded (not recruited or excluded from analysis, see Fig 1). Twenty-eight percent of cases were diagnosed prenatally, 30% before they were one month old, and 42% thereafter. Case mothers were recruited after a diagnosis of CHD from September 2014 to February 2017 [20].

Controls were babies without CHD, recruited at maternity outpatient departments, when attending for a routine fetal anomaly scan at 18–22 weeks gestation in all 17 units across Northern Ireland during the period June 2014 to February 2016. A “one month per unit” approach achieved a representative sample of pregnant women in Northern Ireland [20], with the researcher inviting all eligible women attending for their first anomaly scan during that month in that unit to participate. As shown in Fig 1, twelve control babies subsequently went on to be diagnosed with CHD (approximately the number expected at a rate of CHD in the population of 8 per 1000), and these transferred to case status.

The recruited sample was 286 cases and 966 controls a recruitment rate of 62% and 67% respectively (Fig 1), with refusal rate of 8% for each. 40 cases with a genetic syndrome, and four cases with a minor CHD (patent foramen ovale) were excluded (Fig 1), leaving 242 cases. Only one of the 242 non-genetic cases was a stillbirth and two babies died in the first week of life. 35 cases were born preterm.

Cases were classified according to the information in the paediatric cardiology clinical database by paediatric cardiologists (FC, BC) into one or more of 10 main categories based on anatomical and clinical criteria [21] (Table 1). This classification using the International Paediatric Cardiology Code provides a systematic segmental approach to the description of CHD from systemic venous morphology through to the great arteries which has a strong parallel in embryogenesis. It is shown here to allow the diagnostic spectrum to be compared with other study populations. We do not give results specific to CHD type in this paper due to lack of statistical power but they are available on request.

**Table 1:** Frequency of cases by category of congenital heart defect.

		No*	%
Houyel et al (23) categories			
1	Heterotaxy, including isomerism and mirror-imagery	0	0.0
2	Anomalies of the venous return	6	2.5
3	Anomalies of the atria and interatrial communications (including atrial septal defects)	20	8.3
4	Anomalies of the atrioventricular junctions and valves (including atrioventricular septal defects and ostium primum defects)	11	4.5
5	Complex anomalies of atrioventricular connections	0	0.0
6	Functionally univentricular hearts (including hypoplastic left heart)	24	9.9
7	Ventricular septal defects (VSD)	70	28.9
8	Anomalies of the ventricular outflow tracts (including Tetralogy of Fallot and Transposition of the Great Arteries)	39	16.1
9	Anomalies of the extrapericardial arterial trunks (including Great artery anomalies, including Coarctation of Aorta)	65	26.9
10	Congenital anomalies of the coronary arteries	1	0.4
Missing		6	2.5
Total		242*	

*excluding genetic syndromes; including one case with maternal history of severe CHD.

### Exposure data definition and sources

The exposure period of interest was the periconceptional period, defined as the three months before conception (for longer acting exposures and women’s “normal” behaviours until they recognise their pregnancy) and the first trimester.

Women were asked to complete a self-report questionnaire, available on iPad (at clinic appointments) or in a paper version for postal return (Fig 1) [20]. Mothers of controls completed this at the time of their anomaly scan appointment (median 20 weeks gestation, range 18–34 weeks gestation). Mothers of cases completed this after diagnosis, prenatally (13% of cases) or postnatally (87% of cases) (Fig 1), median 56 weeks after the start of pregnancy.

Information was collected on: maternal and paternal age; maternal education; main home postcode; occupation during the first trimester; previous pregnancy history; pregnancy planning; use of folic acid supplementation in the periconceptional period and consumption of folic acid fortified foods during the first three months of pregnancy (from a list of all available foods in NI at the time which were fortified with folic acid on a voluntary basis); frequency of eating a range of food groups, focusing on food types with high folic acid content, during the three months before pregnancy; smoking during the three months before pregnancy; alcohol consumption in the three months before pregnancy; experience of negative life events (self or someone close) and pregnancy related stress during the periconceptional period; history of chronic health conditions diagnosed by a doctor (self and father of baby); maternal infections and exposure to medications or treatments during the first trimester. For maternal depression and other chronic health conditions, we examined the further details given on the time of diagnosis, and only considered diagnoses in the first trimester or before the index pregnancy.

Data were extracted from the Northern Ireland Maternity System records (NIMATS) recording data collected by the midwife at booking (booking usually occurs at 10–12 weeks gestation); including date of last menstrual period (LMP), previous pregnancy history; weeks gestation at booking; Body Mass Index (BMI); experience of nausea; maternal and paternal smoking at booking; maternal risk factors, mental health and wellbeing in the month prior to booking (two standard questions relating to whether during the past month the woman had experienced symptoms of “feeling down, depressed or hopeless”; or “little interest in or pleasure in doing things”). Women who refused permission to access records (n = 23) or could not be traced on the NIMATS system (n = 6) were treated as missing.

Pregestational diabetes was assessed from the self report questionnaire and verified by prescription records or self declared medication with antidiabetics. Gestational diabetes was as reported in the NIMATS database at any time during pregnancy, excluding any women with pregestational diabetes.

There is high immune status to rubella in the pregnant population and no recent reported cases of congenital rubella. No rubella was reported among maternal risk factors in the maternity data for cases or controls.

We extracted information on medications prescribed during the periconceptional period from the Northern Ireland Enhanced Prescribing Database (EPD), focusing on British National Formulary chapters 3 (Respiratory system); 4 (Central nervous system); 5 (Infections); 6 (Endocrine system) and 9 (Nutrition and blood). Data for women who refused permission to access prescription records (25 controls, 4 cases) or had no available health and care number (4 cases, 5 controls) were treated as missing.

Postcode of residence in the first trimester was linked to the Northern Ireland Multiple Deprivation Measure to obtain the area quintile of socioeconomic deprivation [22].

### Statistical analysis

Crude and adjusted Odds Ratios (OR) with 95% Confidence Intervals (CI) were estimated using unconditional logistic regression, in Stata v12, with listwise deletion. We explored each risk factor/covariate using univariable models with the presence or absence of CHD (case/control status) as the dependent variable. We then constructed multivariate logistic regression models.

In multivariate models, cases and controls with pregestational diabetes were excluded as this is a known strong risk factor for CHD, is associated with some other risk factors, and cannot be successfully adjusted for due to very small numbers affected. We included as covariates sociodemographic factors (maternal age, previous pregnancy, maternal education, socioeconomic deprivation of area of residence) and common risk factors which were potentially associated with CHD according to the literature: folic acid supplementation [7,8], smoking [9,10], BMI category [11–14], antidepressant prescription in first trimester [15–19], dietary class [23–25] (except for analysis of individual food types), pregnancy stress and multiple stressors [26–28]. 9.7% of cases/controls were excluded due to at least one missing value.

We considered that Odds Ratios with a Confidence Interval not including 1 on univariable or multivariate analysis *and* where adjustment for confounders did not greatly shift the point estimate towards 1 constituted supportive evidence for association with CHD.

For medications, we analysed prescriptions issued in the three months following the LMP. Where there was more than 15 days discrepancy between LMP as recorded by NIMATS and other estimates of LMP from self-reported data, the data were inspected for errors and either the most consistent value was chosen (53 controls, 8 cases), or LMP was set to missing (n = 7).

We conducted some chi square analyses (p<0.1) of the association between risk factors in our control population to inform interpretation: the relationship between maternal age and the main risk factors (covariates) as listed above, the relationship between maternal education and the main risk factors, and the relationship between diet and obesity. We did not systematically address interaction between risk factors on risk of CHD, except the hypothesis suggested in the literature [29] of an interaction between smoking and obesity (by computing odds ratio for smoking within overweight/obese subgroup).

Latent Class modelling, using MPlus 6.1, was used to identify groups of women with similar dietary behaviour (a “latent variable”) based on its impact on the frequency of consumption of different foods [30] [31]. A likert scale of frequency of consumption was used, except for liver which was categorised as ever eaten/never eaten. Models ranging from 1 to 6 classes were estimated using Maximum Likelihood estimation with robust standard errors. All thresholds were freely estimated across classes for each food/food group (i.e. no constraints were made on estimated parameters). The optimum number of classes was determined based on a combination of: (i) model fit (the Loglikelihood, Bayesian Information Criterion (BIC), sample size adjusted BIC (SSABIC), Akaike Information Criterion (AIC); (ii), the accuracy with which models classified individuals into their most likely class (Entropy); (iii) a statistical model comparison (Lo-Mendell-Rubin Adjusted Likelihood ratio (LMR)); and (iv) substantive interpretation. The most parsimonious solution was chosen. For each study participant their most likely class membership (based on posterior probabilities), was used in further analyses.

The choice of variables to analyse was as follows:

1. The primary hypotheses concerned folic acid supplements, obesity (BMI), smoking, depression/antidepressants. All related variables were analysed (e.g. diet, stress). All major sociodemographic and reproductive history variables were analysed as descriptive variables and as potential confounders.
2. Exploratory analysis (hypothesis generating analysis) concerned all other recorded exposures where there were at least 3 exposed cases. In multivariate analyses of these variables, we adjusted for the same covariates as selected in the analyses of primary hypotheses. As recommended [32], we did not make multiple comparison adjustments, but interpreted the findings in the light of the multiple comparisons made. Unless the univariate analysis showed statistically significant results, we put these results in S1 to S3 Tables for future meta-analyses.

Information about the dataset and Stata scripts can be found at https://doi.org/10.21251/5b8fabfa-f4c5-465a-ba3d-cadf285313b3.

### Ethics and governance

Ethical approval for the study was obtained in 2014 from the Office for Research Ethics Committees Northern Ireland (ORECNI; 14NI0027) and each Health and Social Care Trust in NI gave Research Governance approval. All participants gave written informed consent, which included discrete consent options to allow access to medical records.

## Results

### Sociodemographic and reproductive characteristics

Cases and controls were similar in maternal age and parity (Table 2). One third had not planned to become pregnant, not differing significantly between cases and controls (Table 2). Cases were more likely than controls to have the lowest level of maternal education (adjOR 1.59, 95%CI 1.02–2.49; Table 2). There was no association with socioeconomic deprivation of area of residence (Table 2).

**Table 2:** Association between maternal risk factors (sociodemographic, reproductive history and diabetes) and congenital heart defects.

	CHD cases (n = 242)		Controls (n = 966)			
	No.	%	No.	%	OR (95%CI)	adjOR* (95%CI)
**Maternal age**						
<25	38	15.7	158	16.4	1.05 (0.68–1.62)	0.66 (0.38–1.14)
25–29	68	28.1	256	26.5	1.16 (0.81–1.67)	0.97 (0.64–1.47)
30–34	80	33.1	350	36.2	Ref	Ref
35+	56	23.1	202	20.9	1.21 (0.83–1.78)	1.17 (0.77–1.79)
**Paternal Age**						
<25	18	7.44	92	9.52	0.76 (0.43–1.34)	0.57 (0.25–1.27)
25–29	51	21.1	231	23.9	0.86 (0.58–1.27)	0.68 (0.41–1.12)
30–34	78	32.2	304	31.5	Ref	Ref
35+	89	36.8	331	34.3	1.05 (0.74–1.47)	1.27 (0.83–1.95)
Missing	6	2.48	8	0.83		
**Maternal education**						
Low (compulsory only)	65	26.9	193	20.0	1.63 (1.13–2.34)	1.59 (1.02–2.49)
Medium (completed high school)	89	36.8	351	36.3	1.22 (0.88–1.70)	1.14 (0.77–1.68)
High (tertiary or other higher)	86	36.3	421	43.6	Ref	Ref
Missing	1	0.42	1	0.10		
**Socioeconomic Deprivation quintile**						
1 (most deprived)	61	25.2	196	20.3	1.23 (0.78–1.96)	1.07 (0.63–1.81)
2	50	20.7	200	20.7	0.99 (0.62–1.60)	0.87 (0.52–1.46)
3	39	16.1	212	22.0	0.73 (0.44–1.20)	0.62 (0.36–1.06)
4	43	17.8	191	19.8	0.89 (0.55–1.46)	0.91 (0.54–1.53)
**Previous pregnancy loss**						
None	160	66.1	627	64.9	Ref	Ref
One	55	22.7	224	23.2	0.96 (0.68–1.35)	0.84 (0.56–1.26)
Two	12	4.96	62	6.42	0.76 (0.40–1.44)	0.69 (0.34–1.41)
Three or more	7	2.89	25	2.59	1.10 (0.47–2.58)	0.81 (0.29–2.30)
Missing	8	3.31	28	2.90		
**Pregnancy Planning**						
Did not plan to become pregnant	89	36.8	320	33.1	1.17 (0.88–1.58)	1.05 (0.70–1.58)
Trying to become pregnant (all other categories)	152	62.8	642	66.5	Ref	Ref
Missing	1	0.41	4	0.41		
**Fertility Clinic (if trying longer than year)**						
Yes	22	59.46	57	50.0	1.45 (0.66–3.19)	2.00 (0.39–10.22)
No	13	35.14	49	43.0	Ref	Ref
Missing	2	5.41	8	7.02		
**Pregestational Diabetes**						
Yes	4	1.65	4	0.41	4.04 (1.00–16.28)	NA
No	238	98.4	962	99.6	Ref	
**Gestational Diabetes (excl pregestational diabetes)**						
Yes	12	8.33	36	3.74	1.30 (0.67–2.55)	NA
No	202	84.9	790	82.1	Ref	
Missing	24	10.1	136	14.1		

*All multivariate models excluded cases/controls with pregestational diabetes and included the following variables: maternal age, previous pregnancy, maternal education, socioeconomic deprivation of area of residence, dietary class, BMI category, folic acid supplementation, smoking, antidepressant prescription in first trimester, pregnancy stress, multiple stressors. N = 1098 for cases/controls non-missing for all these variables and excluding cases/controls with pregestational diabetes.

We examined the relationship of maternal age and education with our main risk factors of interest. In our population, both young maternal age and low maternal education were associated with not taking preconceptional folic acid, poor diet, smoking, and taking antidepressants. Obesity was associated with low maternal education but not maternal age. Multiple stressors were associated with younger maternal age but not education. Pregnancy stress was associated with neither maternal age nor maternal education. Adjusting for these risk factors had little impact on the Odds Ratio for low maternal education (Table 2).

### Maternal diabetes and obesity

Pregestational Diabetes was strongly associated with CHD (OR 4.04, 95%CI 1.00–16.28 Table 2) though infrequent (1.7% of cases). The CHD diagnoses were two VSD, one Transposition of Great Arteries, and one Double Inlet Left Ventricle. Of the eight cases/controls with diabetes, seven had not planned to become pregnant, six had a previous pregnancy, and only two had taken preconceptional folic acid.

Excluding pregestational diabetes, 5.7% of cases and 4.4% of controls were diagnosed with gestational diabetes: OR 1.30, 95%CI 0.67–2.55; (Table 2).

Obesity, based on BMI at booking, was not associated with CHD risk, before or after adjustment for diabetes and other covariates (Table 3).

**Table 3:** Association between maternal risk factors (folic acid, diet, smoking, alcohol, obesity) and congenital heart defects.

	CHD cases (n = 242)		Controls (n = 966)			
	No.	%	No.	%	OR (95%CI)	adjOR* (95%CI)
**Self-report Folic acid Supplement**						
Did not take in first trimester	22	9.09	71	7.4	1.31 (0.77–2.23)	1.10 (0.60–2.01)
Started before pregnancy	93	38.	394	40.8	Ref	Ref
Started after conception and before 6 weeks gestation	61	25.2	277	28.7	0.93 (0.65–1.33)	0.86 (0.57–1.29)
Started between 6–12 weeks gestation	57	23.6	215	22.3	1.12 (0.78–1.62)	1.01 (0.66–1.56)
Missing	9	3.72	9	0.9		
**Preconception Folic acid Supplement (as reported to the midwife)**						
Yes	78	32.2	340	35.2	Ref	Ref
No	110	45.5	518	53.6	0.93 (0.67–1.28)	0.86 (0.60–1.24)
Missing	54	22.3	108	11.2		
**Fortified foods**						
No fortified foods	59	24.4	193	20.0	Ref	
one type fortified food	98	40.5	375	38.8	0.85 (0.59–1.23)	0.82 (0.54–1.23)
two types fortified foods	58	24.0	257	26.6	0.74 (0.49–1.11)	0.78 (0.50–1.22)
three/four types fortified foods	27	11.2	141	14.6	0.63 (0.38–1.04)	0.61 (0.35–1.08)
**Diet**						
Type 1—Moderate Fruit&Veg	80	33.1	378	39.1	Ref	Ref
Type 2—Varied diet- High F&V	77	31.8	326	33.8	1.12 (0.79–1.58)	1.19 (0.81–1.75)
Type 3—Poor diet-Low F&V	85	35.1	260	26.9	1.54 (1.10–2.18)	1.56 (1.05–2.34)
Missing	0	0.00	2	0.21		
**Fizzy or high energy drinks**						
Fizzy or high energy every day	49	20.3	128	13.3	1.67 (1.15–2.42)	1.41 (0.90–2.21)
Fizzy or high energy 3+/wk	36	14.9	151	15.6	1.04 (0.69–1.55)	1.07 (0.68–1.70)
Fizzy or high energy <3/wk	155	64.1	675	69.9	Ref	Ref
Missing	2	0.83	12	1.24		
**Food types eaten 3 or more times per week**						
Broccoli, brussel sprouts, spinach, peas, dark leafy veg (F)	72	29.8	236	24.4	1.30 (0.95–1.78)	1.37 (0.97–1.95)
Raw/lightly cooked veg (F)	48	19.8	245	25.4	0.72 (0.51–1.02)	0.91 (0.63–1.33)
Brown rice, chickpeas, kidney beans, lentils (F)	17	7.02	44	4.6	1.55 (0.87–2.77)	1.75 (0.93–3.31)
Oranges, strawberries, raspberries, pineapple, kiwi, cantaloupe, lemons, limes (F)	100	41.3	503	52.1	0.64 (0.48–0.85)	0.64 (0.47–0.89)
Other fruit (e.g apples, bananas, pears)	148	61.2	630	65.2	0.83 (0.62–1.10)	0.86 (0.62–1.20)
Tomatoes	81	33.5	313	32.4	1.05 (0.78–1.41)	1.06 (0.77–1.47)
Liver (F)	1	0.41	2	0.2		
Other meats	185	76.5	713	73.8	1.11 (0.80–1.55)	1.30 (0.89–1.90)
Processed meats e.g. sausages, bacon	59	24.4	213	22.1	1.12 (0.81–1.57)	1.20 (0.83–1.74)
Fish	17	7.02	59	6.1	1.15 (0.66–2.00)	1.21 (0.66–2.24)
Dairy	217	89.7	856	88.6	1.10 (0.68–1.78)	1.17 (0.68–2.00)
Low calorie	21	8.68	117	12.1	0.68 (0.42–1.10)	0.68 (0.40–1.16)
**Smoking (preconception) (self report)**						
Non smoker	179	74.3	749	77.5	Ref	Ref
Light smoker 1–10 per day	44	18.3	149	15.4	1.24 (0.85–1.80)	1.12 (0.72–1.75)
Heavy smoker 11+ per day	18	7.47	66	6.85	1.14 (0.66–1.97)	0.66 (0.32–1.38)
Missing	1	0.4	2	0.2		
**Smoking (no. cigarettes/day at midwife booking)**						
non-smoker	182	75.2	779	80.6	Ref	Ref
1-10/day	29	12.0	93	9.63	1.33 (0.85–2.09)	1.07 (0.62–1.84)
11+/day	3	1.24	16	1.66	0.80 (0.23–2.78)	0.47 (0.10–2.24)
Missing	28	11.6	78	8.07		
**Smoking (paternal) (no. cigarettes/day at midwife booking)**						
Non smoker	159	65.7	682	70.6	Ref	Ref
Light smoker 1–10 per day	34	14.1	127	13.2	1.14 (0.76–1.74)	1.08 (0.67–1.76)
Heavy smoker 11+ per day	17	7.02	68	7.04	1.07 (0.61–1.88)	0.84 (0.42–1.68)
Missing	32	13.2	89	9.21		
**Alcohol use (preconception) (self report)**						
Not at all or less than once a month	116	47.9	477	49.4	Ref	Ref
once or twice a month	62	25.6	212	21.9	1.20 (0.85–1.70)	1.20 (0.81–1.79)
at least once or twice a week	63	26.0	275	28.5	0.94 (0.67–1.32)	1.05 (0.71–1.54)
missing	1	0.41	2	0.21		
**Obesity (BMI kg/m2)**						
Underweight (<18.50)	3	1.24	11	1.14	1.11 (0.30–4.03)	1.50 (0.39–5.77)
Normal (18.50–24.99)	113	46.7	458	47.4	Ref	Ref
Overweight (25.00–29.99)	72	29.8	274	28.4	1.07 (0.76–1.48)	1.02 (0.71–1.46)
Obese (30.00–39.99)	41	16.9	167	17.3	1.00 (0.67–1.48)	0.98 (0.63–1.51)
Morbidly obese (40.00+)	7	2.89	33	3.42	0.86 (0.37–1.99)	0.70 (0.28–1.81)
Missing	6	2.48	23	2.38		
**Frequency of exercise in first three months of pregnancy**						
Infrequently/not at all	135	55.79	522	54.04	1.02 (0.71–1.48)	1.09 (0.71–1.66)
3/4 times a week	58	23.97	250	25.88	0.92 (0.60–1.40)	1.11 (0.69–1.79)
Every day /nearly every day	49	20.25	194	20.08	Ref	Ref
Missing	0	0	0	0		

* All multivariate models excluded cases/controls with pregestational diabetes and included the following variables: maternal age, previous pregnancy, maternal education, socioeconomic deprivation of area of residence, dietary class (except for analysis of individual food types), BMI category, self reported folic acid supplementation (except when analysing preconceptional folic acid reported to midwife), smoking (except when analysing smoking reported to midwife), antidepressant prescription in first trimester, pregnancy stress, multiple stressors.

### Folic acid and other dietary variables

Most women were taking folic acid by the end of their first trimester (Table 3). None of the measures relating to folic acid supplements (self-reported non-use in first trimester or use after 6 weeks gestation, and not starting supplements preconceptionally as reported to the midwife) were significantly associated with CHD risk (Table 3).

Three quarters of the women stated they were consuming at least one type of food (cereal, cereal bars, breads or spreads) fortified with folic acid (Table 3). There was a non-significant trend for the number of types of fortified foods consumed to be associated with a lowering of CHD risk.

**Fig 2:**
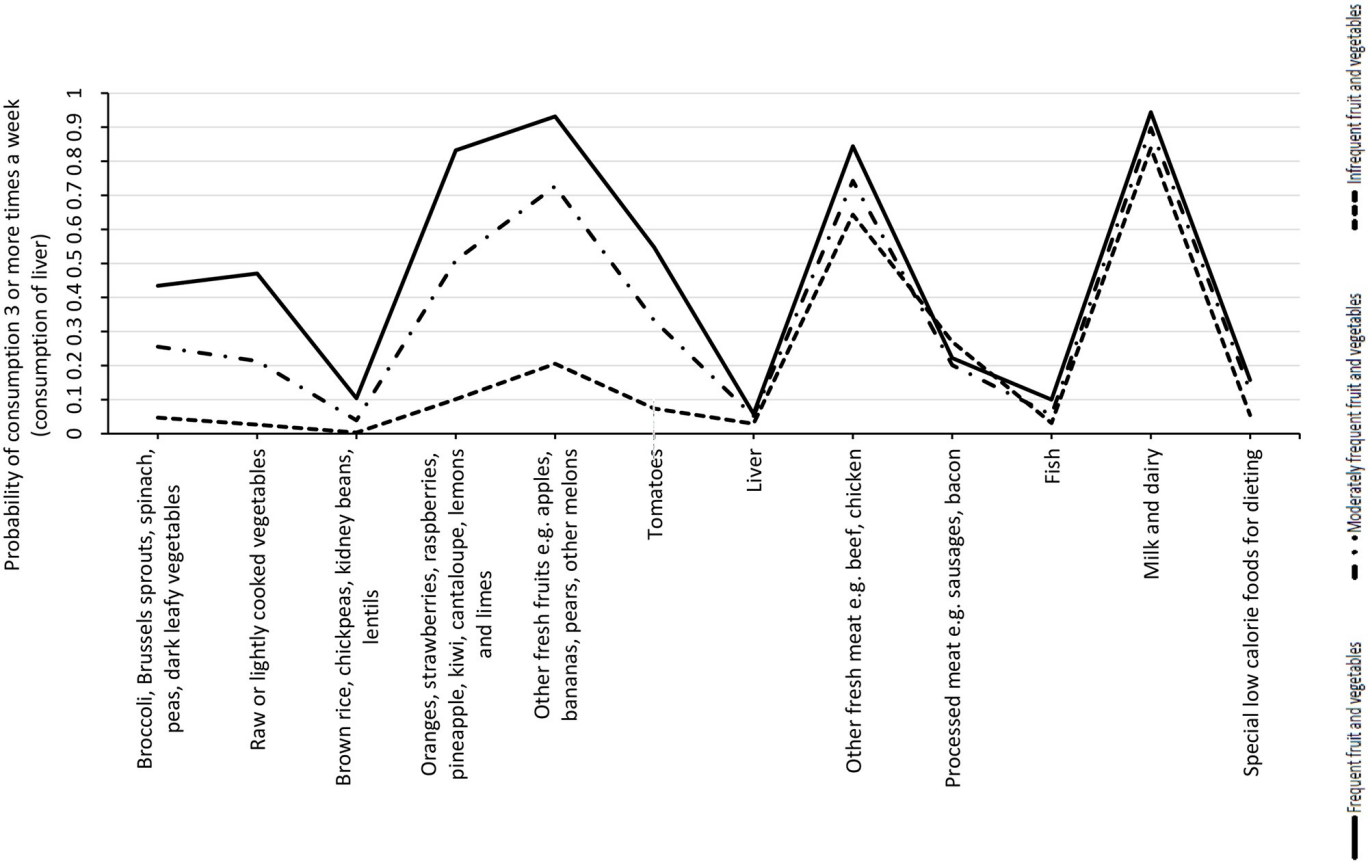
Estimated probabilities of eating the different food types 3 or more times per week (liver: Eaten/not eaten), in each dietary class.

Three dietary classes were delineated (Fig 2): “Moderate Fruit and Vegetable” (38% of the study population), “Varied Diet” in particular with the highest fruit and vegetable consumption (33.4%) and “Poor Diet” in particular with low fruit and vegetable consumption (28.6%). Compared to the largest dietary group, a higher risk was found for “poor diet” (adjOR 1.56, 95%CI 1.05–2.34; Table 3). Diet was not significantly associated with obesity in our population (p = 0.92).

Analysing individual diet components, eating oranges and other folate rich fruits 3 times a week or more was associated with a significantly lower risk (OR 0.64, 95%CI 0.47–0.89).

Drinking fizzy or high energy drinks *every* day was significantly associated with CHD in univariable analysis (OR 1.67, 95%CI 1.15–2.42) but this attenuated with adjustment for covariates (adjOR 1.41, 95%CI 0.90–2.21, Table 3) since daily consumption of fizzy drinks was associated with factors such as poor diet and low education. Inspection of data revealed no discernible differences in risk between fizzy and high energy drinks but numbers were small.

### Maternal smoking

Neither self-reported smoking before pregnancy, nor smoking as reported to the midwife at booking, were significantly associated with the risk of CHD (Table 3). Subgroup analysis focusing on the subgroup of obese or overweight women did not find self-reported smoking to be a significant risk factor (light smokers adjOR 0.89, 95%CI 0.47–1.69, heavy smokers adjOR 0.58, 95%CI 0.22–1.56).

### Maternal mental health, mental health related medication, and stress

None of the measures of maternal depression were significantly or strongly associated with CHD. Odds ratios attenuated considerably after adjustment for confounders (Table 4). Antidepressant use, whether self-reported or based on prescription data, was not associated with CHD risk.

**Table 4:** Association between maternal mental health—Associated risk factors (maternal mental health conditions, stress, and associated medications) and congenital heart defects.

	CHD cases (n = 242)		Controls (n = 966)			
	No.	%	No.	%	OR (95%CI)	adjOR* (95%CI)
**Ever Diagnosed with depression (self-report)**						
No	201	83.1	838	86.8	Ref	Ref
Yes	41	16.9	128	13.3	1.33 (0.91–1.96)	0.99 (0.60–1.65)
**Ever Diagnosed with anxiety (self-report)**						
No	213	88.0	850	88.0	Ref	Ref
Yes	29	12.0	116	12.0	1.00 (0.65–1.54)	0.90 (0.54–1.51)
**Ever diagnosed Other mental health conditions (self-report)**						
No	236	97.5	949	98.2	Ref	Ref
Yes	6	2.48	17	1.76	1.42 (0.55–3.64)	1.43 (0.48–4.26)
**Last month depressed (at midwife booking)**						
No	203	83.9	855	88.5	Ref	Ref
Yes	9	3.72	19	1.97	2.00 (0.89–4.47)	1.20 (0.45–3.20)
Missing	30	12.4	92	9.52		
**Last month little interest (at midwife booking)**						
No	205	84.7	858	88.8	Ref	Ref
Yes	7	2.89	16	1.66	1.83 (0.74–4.51)	1.43 (0.52–3.97)
Missing	30	12.4	92	9.52		
**Antidepressants (any type) in first trimester (self-report)**						
No	235	97.1	932	96.5	Ref	Ref
Yes	7	2.89	34	3.52	0.82 (0.36–1.87)	0.73 (0.29–1.82)
Antidepressants—SSRI/SNRI in first trimester (prescription data)**						
No	215	88.8	882	91.3	Ref	Ref
Yes	13	5.37	51	5.28	1.04 (0.56–1.96)	0.90 (0.44–1.83)
Missing	14	5.79	33	3.42		
**Counselling/ behaviour therapy (self-report)**						
No	235	97.1	952	98.6	Ref	Ref
Yes	7	2.89	14	1.45	2.03 (0.81–5.07)	1.89 (0.64–5.57)
Missing	0	0	0	0.00		
Stressful events in periconceptional period (Self-report)***						
Death	29	12.0	101	10.4		
Family ill	20	8.3	86	8.9		
Move house	25	10.3	104	10.8		
Lost job	13	5.4	39	4.0		
Relation diffs	13	5.4	23	2.4		
Alco abuse	8	3.3	16	1.7		
Discrimination	3	1.2	7	0.7		
Legal probs	7	3.0	14	1.5		
Victim of crime	3	1.2	10	1.0		
Arrest	5	2.1	6	0.6		
Stressful events combined						
None	159	65.7	673	69.7	Ref	Ref
<3	72	29.8	271	28.1	1.12 (0.82–1.54)	1.10 (0.78–1.57)
3+	11	4.55	22	2.28	2.12 (1.01–4.45)	1.94 (0.83–4.53)
**Pregnancy related stress (Self-report)**						
No	129	53.3	658	68.1	Ref	Ref
Yes	112	46.3	307	31.8	1.86 (1.40–2.48)	1.69 (1.22–2.34)
Missing	1	0.41	1	0.10		
**Perceived social support (Self-report)**						
No	12	4.96	25	2.59	1.98 (0.98–3.99)	1.51 (0.63–3.62)
Yes	228	94.2	939	97.2	Ref	Ref
Missing	2	0.83	2	0.21		

* All multivariate models excluded cases/controls with pregestational diabetes and included the following variables: maternal age, previous pregnancy, maternal education, socioeconomic deprivation of area of residence, dietary class, BMI category, folic acid supplementation, smoking, antidepressant prescription in first trimester (except for analysis of self reported antidepressants), pregnancy stress, multiple stressors.

**Prescribed SSRIs in the first three months of pregnancy were Citalopram, Escitalopram, Fluoxetine, and Sertraline; prescribed SNRI were Mirtazapine and Venlafaxine.

*** We did not test stressors individually due to small numbers and lack of a theoretical basis for distinguishing individual stressors in this context.

Pregnancy-related stress was associated with CHD (adjOR1.69, 95%CI 1.22–2.34) and was the most common form of stress (reported by 46% of cases and 32% of controls). Multiple stress (the experience of three or more stressful events other than pregnancy related stress) in the periconceptional period, was associated with CHD on univariable analysis (OR 2.12, 95%CI 1.00–4.45), but was slightly attenuated controlling for pregnancy stress and other confounding variables (adjOR 1.94, 95%CI 0.83–4.53). A perceived lack of social support during the first trimester of pregnancy from family and friends was non-significantly associated with CHD (OR 1.98, 95%CI 0.98–3.99, adjOR 1.51, 95%CI 0.63–3.62), as was having counselling/behaviour therapy in the first trimester (adjOR 1.89, 95%CI 0.64–5.57; Table 4).

### Exploratory analysis of other risk factors

Odds ratios for CHD in relation to risk factors expected to be less common in our population, or which did not constitute primary hypotheses based on previous literature, can be found in the S1 to S3 Tables. Significant findings and related variables are shown in Table 5.

**Table 5:** Exposures showing statistically significant associations with CHD on exploratory analysis, and related exposures (full results in Appendix).

	CHD cases (n = 242)		Controls (n = 966)			
	No.	%	No.	%	OR (95%CI)	adjOR* (95%CI)
**Maternal Clotting disorder (self report)**						
No	238	98.3	964	99.8	Ref	Ref
Yes	4	1.65	2	0.21	8.10 (1.48–45.49)	9.69 (1.64–57.4)
**Maternal Heart Disease (self report)**						
No	239	98.8	963	99.7	Ref	Ref
Yes*	3	1.24	3	0.31	4.03 (0.81–20.09)	2.13 (0.34–13.39)
**Maternal raised blood pressure (self report)**						
No	236	97.52	943	97.6	Ref	Ref
Yes	6	2.48	23	2.38	1.04 (0.42–2.59)	1.04 (0.37–2.92)
**Paternal raised blood pressure (maternal report)**						
No	216	89.3	920	95.2	Ref	Ref
Yes	10	4.13	13	1.35	3.28 (1.42–7.57)	2.59 (1.00–6.74)
Missing	16	6.61	33	3.42		
**Vaginal thrush/infection**						
No	212	87.6	892	92.34	Ref	Ref
Yes	30	12.4	74	7.66	1.71 (1.09–2.67)	1.69 (1.01–2.80)
**Enoxaparin (BNF 2.8.1): Prescription in first three months**						
No	228	94.2	928	96.1	Ref	
Yes	6	2.48	8	0.83	3.05 (1.05–8.89)	2.90 (0.95–8.81)
Missing	8	3.31	30	3.11		
**Aspirin (BNF 2.9.0): Prescription in first three months**						
No	218	90.1	904	93.6	Ref	Ref
Yes	16	6.61	32	3.31	2.07 (1.11–3.85)	1.73 (0.86–3.47)
Missing	8	3.31	30	3.11		

*including two controls with maternal congenital heart defects

Self-reported maternal clotting disorder showed a significant relationship with CHD (adjOR 9.69, 95%CI 1.64–57.4, based on 4 exposed cases and 2 exposed controls; Table 5). Prescriptions in the first three months of pregnancy of the anti-clotting medication enoxaparin was associated with CHD (enoxaparin: OR 3.05, 95%CI 1.05–8.89; adjOR 2.90, 95%CI 0.95–8.81; Table 5), and to a lesser extent aspirin (OR 2.07, 95%CI 1.11–3.85, adjOR 1.73, 95%CI 0.86–3.47; Table 5).

The significant enoxaparin finding was further explored in relation to indication, dose, and timing of exposure. Of the 14 women (cases/controls) receiving enoxaparin, two reported a clotting disorder, and seven others reported two or more previous pregnancy losses, one of the indications of enoxaparin treatment. After adjustment for previous pregnancy loss (not in itself a significant risk factor; Table 2) in addition to other confounders, the enoxaparin odds ratio changed little (adjOR 3.22, 95%CI 1.01–10.22) Of the 14 women receiving enoxaparin, 13 had a daily dose of 40 mg and one a daily dose of 60 mg; 5 were coprescribed aspirin at 75mg per day. Seven of the fourteen started their prescriptions in the first six weeks of pregnancy (one preconceptional, six at 4–5 weeks gestation), all but one of whom had multiple previous pregnancy losses. Early exposure (in first 6 weeks) was more common for CHD cases: 5 of 6 cases vs 2 of 8 controls, an OR of 10.17 (95%CI 1.96–52.74).

Paternal raised blood pressure was significantly associated with CHD (adjOR 2.59, 95%CI 1.00–6.74; Table 5) but not maternal raised blood pressure (adjOR 1.04, 95%CI 0.37–2.92). Maternal heart disease showed a non-significantly elevated risk (adjOR 2.13, 95%CI 0.34–13.39; Table 5).

Vaginal infection or thrush was significantly associated with CHD (adjOR 1.69, 95%CI 1.01–2.80; Table 5).

Self-reported use of thyroid medication and prescribed levothyroxine during the first trimester was non-significantly associated with an increased risk of CHD (self report: adjOR 1.70, 95%CI 0.75–3.89; prescription: adjOR 1.92, 95%CI 0.79–4.67; S2 Table). Other exposures were not strongly or significantly associated with CHD, including nausea/vomiting, anti-nausea medication, fever, influenza, kidney infection, self- reported asthma, and self- reported anaemia (S1 and S2 Tables). Antibiotics were not significantly associated with CHD risk but raised OR over 2 were observed for clarithromycin related and nitrofurantoin antibiotics. Self-reported regular use of painkillers was associated with a protective effect (adjOR 0.29, 95%CI 0.08–0.97).

Two common maternal occupations with significant chemical exposures, hairdresser and cleaner, were not strongly associated with CHD (S3 Table).

## Discussion

### Sociodemographic factors

We found an excess risk of CHD associated with low maternal education, which persisted after adjusting for other risk factors, suggesting that unmeasured environmental factors which associate with maternal education (perhaps via individual socioeconomic status) are relevant and need further research. Studies in the UK [33] and Canada [34] have found higher risk of CHD associated with low socioeconomic level of area of residence, which we did not find. CHD may constitute another dimension of socioeconomic inequalities in reproductive health.

### Maternal disease

The fourfold risk of CHD associated with pregestational diabetes in our study is consistent with the literature about this known causal factor, which is associated with poor periconceptional glycaemic control [2,35]. Most of the women with diabetes in our study had not planned to become pregnant, and thus were not accessing preconceptional care to improve glycaemic control and reduce CHD risk, an area of healthcare that needs improvement. We found a low and non-significant risk of CHD associated with gestational diabetes, diagnosed in 4% of pregnancies during this period. A systematic review of the evidence [36] found that gestational diabetes (which occurs after cardiac morphogenesis is complete) was only associated with CHD in the presence of obesity, and concluded this was likely to indicate undiagnosed pregestational diabetes.

Obesity has been associated with a small excess risk of CHD between 10% and 30% [14] [13]. This is not supported by our estimate of risk although within the confidence limits (OR 1.01, 95%CI 0.65–2.06). It is possible that the lack of association with obesity is a chance finding, but other explanations can also be considered. The high prevalence of obesity (BMI 30+) in our population—one quarter of women at booking with midwife–may be associated with types of obesity less associated with metabolic syndrome and therefore less implicated in CHD aetiology. A second possible explanation is that it is not obesity itself but the associated dietary factors that are important. In our population, obesity was not associated with dietary class as we measured it, but in other populations the relationship with diet may differ [12]. The association with obesity in other studies may have been inflated because of bias in measurement between cases and controls (in our study we used the same prospective measurement for cases and controls thus reducing the opportunity for bias), or lack of control for confounding by pregestational diabetes. Bias in other studies may also be caused by poor diagnostic data on CHD resulting in transitory cardiac conditions associated with preterm birth (which is itself related to obesity) being included as CHD. Our study excluded small transitory ASDs as well as, for preterm births, patent ductus arteriosus. A large Swedish study [11] based on record linkage and finding a 12% obesity-related excess of CHD recorded a prevalence of CHD of 1.6% of births, twice the prevalence expected, suggesting inclusion of transitory forms.

A potentially important new finding from the hypothesis-generating part of our study was the threefold risk for CHD among women who were prescribed the anti-clotting medication enoxaparin (adjOR 3.22, 95%CI 1.01–10.22), and women who self-reported blood clotting disorders (adjOR 9.69, 95%CI 1.64–57.38). In our exploratory analysis of many risk factors, some chance findings are likely, but we consider that these two apparently related findings are of particular interest for independent confirmation in other study populations. The enoxaparin risk was more highly associated with being prescribed the medication early in the first trimester (before 6 weeks gestation), consistent with a causal relationship. Enoxaparin is recommended in case of recurrent *consecutive* pregnancy loss [37] on the basis that this is often associated with abnormal blood clotting. There is no previous evidence of elevated malformation risk associated with enoxaparin but there is concern that the evidence base is so limited [38]. Enoxaparin does not cross the placenta and therefore is a preferred alternative to warfarin, a known teratogen, in case of pregnancy. If our findings are confirmed, further research is needed to disentangle the effects of the medication and the possible underlying indication of an undiagnosed blood clotting disorder or other factor associated with recurrent consecutive pregnancy loss. We did not find a general association between CHD and multiple pregnancy loss, but did not have a measure of recurrent consecutive loss. Recurrent pregnancy loss is believed to be associated with heritable thrombophilias (an abnormality of blood coagulation) which lead to impaired placental development and function [39]. If the underlying indication(s) are causing the elevated CHD risk, then our data suggest that enoxaparin treatment in current practice does not eliminate this risk or may be associated with CHD via prevention of miscarriage.

We found a significant association with vaginal infection (adjOR 1.69, 95%CI 1.01–2.80) which has not been previously reported. Previous studies have reported an association between CHD and influenza [40] or fever [41], particularly in the absence of multivitamin use [41]. We could find no evidence of an effect of fever or influenza.

### Folic acid and diet

In our population, the vast majority of women were taking folic acid supplements by the end of the first trimester. We did not find any excess risk of CHD in the small group of women who did not take supplements at all, or among those starting them after 6 weeks gestation. It has been suggested that varying results of studies in different populations may relate to the background folate/folic acid intake of the population [7,8]. In the UK, there is no mandatory food fortification with folic acid, but voluntary food fortification reached three quarters of our population. There was some indication in our data that eating more fortified foods conferred a protective effect, but this was not statistically significant. Frequent consumption of folate rich fruits conferred a protective effect, but needs to be considered within the entire dietary context.

Our findings support a role for maternal diet in the aetiology of CHD. A poor diet, low in fruit and vegetables and many of the other foods measured, was associated with a 56% excess risk of CHD in our population compared to the most common dietary category. This type of study cannot identify which dietary components are causally associated, and the possibility of unmeasured confounding cannot be excluded, but our results do indicate the need for further research on diet. Other studies have found a protective effect of better maternal diet quality in the year preceding pregancy for conotruncal defects [8], a protective effect of a ‘prudent diet’ (high consumption of fruits, vegetables, whole wheat grains, reduced-fat dairy and fish) limited to folic acid supplement users (23), a protective effect of high intakes of fish and seafood [24], and a role for a range of nutrients beyond folate in relation to risk of Tetralogy of Fallot and d-Transposition of the Great Arteries [25].

We found an association with daily consumption of fizzy (“Soda” or carbonated) and high energy drinks, but this was attenuated when controlling for confounding factors such as diet and maternal education (adjOR 1.41, 95%CI 0.90–2.21). We believe our study to be the first to have investigated this. There is evidence that sugar or glucose levels in blood may be related to CHD risk [42] [43] even among non-diabetics. On the other hand, sugar substitutes in diet fizzy/soda drinks have been associated with a risk of diabetes [44,45]. Further research should therefore differentiate sugar and sugar substitute drinks, and include dietary sources of sugar which we did not assess.

### Maternal smoking

Two meta-analyses of studies of smoking and CHD have estimated a 9–11% excess risk among smokers, but without controlling for confounders [9,10]. Our study was not statistically powerful enough to support or reject such a small excess risk, but our data do concur with the general conclusion that a risk, if present, is small, and may depend on interaction with other factors, for example those which affect hypoxia or maternal dyslipidaemia [29] or genetic variants [6]. We did not find an interaction with obesity as previously suggested [29]. Because of the strong relationship between smoking and education (and other sociodemographic factors), and between education and CHD, meta-analyses that do not control for confounding may inflate estimates of risk.

### Maternal mental health and related medication

We did not find an association between CHD risk and maternal use of antidepressants. While meta-analyses [17,18,46,47] find an overall association between CHD risk and all or selected SSRIs (particularly paroxetine which was not used in our population in pregnancy), with pooled odds ratios generally lower than 1.5, continuing debate concerns whether this is due to confounding by depression, co-medications or other co-exposures [19,48]. There may also be a role for genetic polymorphisms in population variation [49]. Our finding that maternal depression itself was not a significant risk factor for CHD adds to a very small evidence base on this question [50], and moreover our results suggest that there is considerable potential for confounding in any association of depression with CHD, since point estimates of odds ratios shifted considerably towards 1 after adjustment.

We did find CHD to be significantly associated with pregnancy stress in the first trimester, which was very commonly reported (32% of controls) and therefore potentially important in public health terms. There was also some evidence that multiple stressful events experienced in the periconceptional period were also associated with CHD. Stress was retrospectively reported and may have been perceived differently by mothers of cases (who had subsequently also experienced the stress of their baby being diagnosed with CHD) and controls. However, our findings are supported by other studies, both studies based like ours on retrospective report of a variety of stressful life events [26], and studies free of recall issues based on linkage with data on death or serious illness of family members [27,28]. The biological mechanism has been hypothesised to be via hyperglycaemia or hypoxia through increased secretion of cortisone and catecholamines [27].

### Strengths and limitations

The strengths of our study were that we were able to conduct a population-based assessment in a geographical area served by a single diagnostic centre; we included well validated diagnoses of CHD, and excluded all genetic syndromes, patent ductus arteriosus associated with preterm birth, and small ASDs which need follow-up to distinguish from patent foramen ovale. We had a rich dataset in terms of potential risk factors and confounders. We used a confidential user-friendly iPad questionnaire and linked the self-reported data to prospective information from maternity records and prescription records to limit recall bias. We have analysed elsewhere [20] our recruitment and found our study sample to be representative of the population, with little evidence of selection bias. Our refusal rates were low and most non-recruitment was because of logistic reasons unrelated to potential risk factors. We have also analysed discordance between prescription data and self-reported data for medications, and found no evidence of bias, but discordance around reporting of very early pregnancy exposures [20] which varies by type of medication.

The main limitation of our study was that we did not achieve our target sample size for cases [20] and therefore had limited statistical power—confidence intervals for less common exposures were wide. it is possible that risk factors for specific CHD subtypes were masked by studying CHD as a whole, and it was not possible to explore possible interactions between exposures of interest. Our App tool is available for use by other studies and we see the current study as a “seed” for further population-based studies in Europe which are sadly lacking.

The second major limitation of our study was that case mothers were interviewed later than control mothers, so they had a longer period of recall, which may also have been affected by intervening events. We have analysed this [20] and cannot find evidence of significant recall bias, but it must nevertheless be recognised as a possibility for some exposures.

We excluded genetic cases diagnosed according to current UK health service practice for CHD (referred for genetic testing due to the presence of dysmorphism, developmental delay or multiple malformations). Some genetic cases may have remained in the data, and this would tend to dilute odds ratios for environmental risk factors towards one. It is also possible that a few cases of CHD may have been attributed to genetic causes despite an environmental component to their aetiology. We may have underascertained stillborn babies with CHD and those who died early, due to the added difficulty of recruitment [20].

We studied maternal risk factors, and included paternal risk factors mainly for their value in relieving potential maternal guilt among respondents, rather than their value as genetic, epigenetic [51] or environmental risk factors. Future research could pay more attention to paternal factors.

## Conclusions

Our results point to an unrealised potential for primary prevention of CHD by the modification of risk factors such as maternal education, diet, maternal stress, and control of diabetes and other maternal diseases. To realise this potential, further research is needed. Prevention of CHD is likely to lead also to more general reproductive health improvement. On the other hand, mothers of children with a congenital heart defect can generally be reassured that many common exposures conferred no risk or a small risk, and that it is more likely that a combination of genetic and environmental factors, many as yet unknown, act together to disrupt fetal heart development.

After a successful phase of improving survival and quality of life for children with CHD, primary prevention is the next barrier to be surmounted.

## Supporting information

S1 File Strobe Checklist. (DOCX)

S1 Table Maternal chronic conditions diagnosed by a doctor and maternal infections in the first three months (maternal self-report), nausea (maternal self-report to midwife at booking). (DOCX)

S2 Table Self-report use and prescribed medication during the first three months. (DOCX)

S3 Table Maternal occupation. (DOCX)
