# Supplementary material for: Risk factors for congenital heart disease: The Baby Hearts Study, a population-based case-control study
Source: PLoS One. 2020 Feb 24;15(2):e0227908. doi: 10.1371/journal.pone.0227908 (PMC7039413; doi:10.1371/journal.pone.0227908)
Supplement: S1 Table — (DOCX) [file pone.0227908.s002.docx]

**S1 Table:** **Maternal chronic conditions diagnosed by a doctor and maternal infections in the first three months (maternal self-report), nausea (maternal self-report to midwife at booking)**

|  | **CHD cases (n= 242 )** | | **Controls (n=966 )** | |  |  |  |  |
| --- | --- | --- | --- | --- | --- | --- | --- | --- |
|  | **No.** | **%** | **No.** | **%** |  |  | **OR (95%CI)** | **adjOR*** |
| **Asthma** |  |  |  |  |  |  |  |  |
| No | 202 | 83.5 | 834 | 86.3 |  |  | Ref | Ref |
| Yes | 40 | 16.5 | 132 | 13.7 |  |  | 1.25 (0.85-1.84) | 1.27 (0.82-1.96) |
|  |  |  |  |  |  |  |  |  |
| **Anaemia** |  |  |  |  |  |  |  |  |
| No | 217 | 89.7 | 882 | 91.3 |  |  | Ref | Ref |
| Yes | 25 | 10.3 | 84 | 8.70 |  |  | 1.26 (0.78-2.02) | 0.99 (0.57-1.70) |
|  |  |  |  |  |  |  |  |  |
| **Kidney Infection** |  |  |  |  |  |  |  |  |
| No | 221 | 91.3 | 902 | 93.4 |  |  | Ref | Ref |
| Yes | 21 | 8.68 | 64 | 6.63 |  |  | 1.34 (0.80-2.24) | 1.07 (0.58-1.97) |
|  |  |  |  |  |  |  |  |  |
| **Vaginal/thrush infection** |  |  |  |  |  |  |  |  |
| No | 212 | 87.6 | 892 | 92.3 |  |  | Ref | Ref |
| Yes | 30 | 12.4 | 74 | 7.66 |  |  | 1.71 (1.09-2.67) | 1.69 (1.01-2.80) |
|  |  |  |  |  |  |  |  |  |
| **Fever first trimester** |  |  |  |  |  |  |  |  |
| No | 223 | 92.2 | 891 | 92.2 |  |  | Ref | Ref |
| Yes | 17 | 7.02 | 73 | 7.56 |  |  | 0.93 (0.54-1.61) | 0.63 (0.32-1.25) |
| Missing | 2 | 0.83 | 2 | 0.21 |  |  |  |  |
|  |  |  |  |  |  |  |  |  |
| **Fever due to influenza first trimester** |  |  |  |  |  |  |  |  |
| No | 233 | 96.3 | 940 | 97.3 |  |  | Ref | Ref |
| Yes | 7 | 2.89 | 24 | 2.48 |  |  | 1.08 (0.46-2.53) | 0.77 (0.26-2.30) |
| Missing | 2 | 0.83 | 2 | 0.21 |  |  |  |  |
|  |  |  |  |  |  |  |  |  |
| **Nausea/vomiting** |  |  |  |  |  |  |  |  |
| None | 48 | 19.8 | 184 | 19.1 |  |  | 1.06 (0.72-1.56) | 1.03 (0.67-1.60) |
| Nausea only | 99 | 40.9 | 403 | 41.7 |  |  | Ref | Ref |
| Occasional vomiting | 33 | 13.6 | 140 | 14.5 |  |  | 0.96 (0.62-1.49) | 0.97 (0.60-1.57) |
| Vomiting most days | 11 | 4.55 | 85 | 8.80 |  |  | 0.53 (0.27-1.02) | 0.45 (0.21-0.99) |
| Vomiting everyday | 19 | 7.85 | 70 | 7.25 |  |  | 1.10 (0.64-1.92) | 1.16 (0.64-2.12) |
| Severe vomiting | 2 | 0.83 | 4 | 0.41 |  |  | 2.03 (0.37-11.27) | 1.79 (0.30-10.71) |
| Missing | 30 | 12.4 | 80 | 8.28 |  |  |  |  |
