# Supplementary material for: Risk factors for congenital heart disease: The Baby Hearts Study, a population-based case-control study
Source: PLoS One. 2020 Feb 24;15(2):e0227908. doi: 10.1371/journal.pone.0227908 (PMC7039413; doi:10.1371/journal.pone.0227908)
Supplement: S2 Table — (DOCX) [file pone.0227908.s003.docx]

**S2 Table: Self-report use and prescribed medication during the first three months**

|  | **CHD cases (n= 242 )** | | **Controls (n=966 )** | |  |  |  |  |
| --- | --- | --- | --- | --- | --- | --- | --- | --- |
|  | **No.** | **%** | **No.** | **%** |  |  | **OR (95%CI)** | **adjOR*** |
| **Thyroid medication (self-report** |  |  |  |  |  |  |  |  |
| **No** | 234 | 97.7 | 948 | 98.1 |  |  | Ref | Ref |
| **Yes** | 8 | 3.31 | 18 | 1.86 |  |  | 1.80 (0.77 – 4.19) | 1.92 (0.79 – 4.67) |
|  |  |  |  |  |  |  |  |  |
| **Levothyroxine 6.2.1 Prescription in first three months** |  |  |  |  |  |  |  |  |
| **No** | 225 | 93.0 | 913 | 94.5 |  |  | Ref | Ref |
| **Yes** | 9 | 3.72 | 23 | 2.38 |  |  | 1.59 (0.72 – 3.48) | 1.70 (0.75 – 3.89) |
| **Missing** | 8 | 3.31 | 30 | 3.11 |  |  |  |  |
|  |  |  |  |  |  |  |  |  |
| **Pain killers regular (self-report)** |  |  |  |  |  |  |  |  |
| No | 238 | 98.4 | 929 | 96.2 |  |  | Ref | Ref |
| Yes | 4 | 1.65 | 37 | 3.83 |  |  | 0.42 (0.15-1.20) | 0.29 (0.08-0.97) |
|  |  |  |  |  |  |  |  |  |
| **Pain killers Few (self-report)** |  |  |  |  |  |  |  |  |
| No | 154 | 63.6 | 592 | 61.2 |  |  | Ref | Ref |
| Yes | 88 | 36.4 | 374 | 38.7 |  |  | 0.90 (0.68-1.21) | 0.88 (0.64-1.22) |
|  |  |  |  |  |  |  |  |  |
| **Antihistamine Few (self-report)** |  |  |  |  |  |  |  |  |
| No | 239 | 98.8 | 935 | 96.8 |  |  | Ref | Ref |
| Yes | 3 | 1.24 | 31 | 3.21 |  |  | 0.38 (0.11-1.25) | 0.30 (0.07-1.29) |
|  |  |  |  |  |  |  |  |  |
| **Antinausea 4.6.0 (Prescription)** |  |  |  |  |  |  |  |  |
| No | 202 | 83.5 | 819 | 84.8 |  |  | Ref | Ref |
| Yes | 32 | 13.2 | 117 | 12.1 |  |  | 1.11 (0.73-1.69) | 1.11 (0.71-1.73) |
| Missing | 8 | 3.31 | 30 | 3.11 |  |  |  |  |
|  |  |  |  |  |  |  |  |  |
| **Antibiotics (Self report)** |  |  |  |  |  |  |  |  |
| No | 215 | 88.8 | 847 | 87.7 |  |  | Ref | Ref |
| Yes | 27 | 11.2 | 119 | 12.3 |  |  | 0.89 (0.57-1.39) | 0.89 (0.53-1.47) |
| Missing | 0 | 0 | 0 | 0 |  |  |  |  |
|  |  |  |  |  |  |  |  |  |
| **Antibiotics**  **(Prescription)** |  |  |  |  |  |  |  |  |
| **Amoxicillin and related 5.1.1** |  |  |  |  |  |  |  |  |
| No | 214 | 88.4 | 838 | 86.8 |  |  | Ref | Ref |
| Yes | 20 | 8.26 | 98 | 10.1 |  |  | 0.80 (0.48-1.32) | 0.79 (0.45-1.38) |
| Missing | 8 | 3.31 | 30 | 3.11 |  |  |  |  |
|  |  |  |  |  |  |  |  |  |
| **Cefalexin and related 5.1.2** |  |  |  |  |  |  |  |  |
| No | 231 | 95.5 | 908 | 94.0 |  |  | Ref | Ref |
| Yes | 3 | 1.24 | 28 | 2.90 |  |  | 0.42 (0.13-1.40) | 0.49 (0.15-1.68) |
| Missing | 8 | 3.31 | 30 | 3.11 |  |  |  |  |
| **Clarithromycin and related 5.1.5** |  |  |  |  |  |  |  |  |
| No | 231 | 95.5 | 929 | 96.2 |  |  | Ref | Ref |
| Yes | 3 | 1.24 | 7 | 0.72 |  |  | 1.72 (0.44-6.72) | 2.21 (0.53-9.28) |
| Missing | 8 | 3.31 | 30 | 3.11 |  |  |  |  |
|  |  |  |  |  |  |  |  |  |
| **Trimethoprim and related 5.1.8** |  |  |  |  |  |  |  |  |
| No | 231 | 95.5 | 928 | 96.1 |  |  | Ref | Ref |
| Yes | 3 | 1.24 | 8 | 0.83 |  |  | 1.51 (0.40-5.72) | 1.04 (0.21-5.10) |
| Missing | 8 | 3.31 | 30 | 3.11 |  |  |  |  |
|  |  |  |  |  |  |  |  |  |
| **Nitrofurantoin 5.1.13** |  |  |  |  |  |  |  |  |
| No | 228 | 94.2 | 924 | 95.7 |  |  | Ref | Ref |
| Yes | 6 | 2.48 | 12 | 1.24 |  |  | 2.03 (0.75-5.46) | 2.13 (0. 70-6.43) |
| Missing | 8 | 3.31 | 30 | 3.11 |  |  |  |  |
|  |  |  |  |  |  |  |  |  |
| **Anti-asthmatic medication (self-report)** |  |  |  |  |  |  |  |  |
| No | 229 | 94.6 | 905 | 93.7 |  |  | Ref | Ref |
| Yes | 13 | 5.37 | 61 | 6.31 |  |  | 0.84 (0.45-1.54) | 0.79 (0.40-1.57) |
| Missing | 0 | 0 | 0 | 0 |  |  |  |  |
| **Adrenoceptor Agonists 3.1.1** |  |  |  |  |  |  |  |  |
| No | 223 | 92.2 | 890 | 92.1 |  |  | Ref |  |
| Yes | 11 | 4.55 | 46 | 4.76 |  |  | 0.95 (0.49-1.87) | 0.92 (0.45-1.90) |
| Missing | 8 | 3.31 | 30 | 3.38 |  |  |  |  |
