# Supplementary material for: Risk factors for congenital heart disease: The Baby Hearts Study, a population-based case-control study
Source: PLoS One. 2020 Feb 24;15(2):e0227908. doi: 10.1371/journal.pone.0227908 (PMC7039413; doi:10.1371/journal.pone.0227908)
Supplement: S3 Table — (DOCX) [file pone.0227908.s004.docx]

**S3 Table: Maternal occupation**

|  | **CHD cases (n= 242 )** | | **Controls (n=966 )** | |  |  |  |  |
| --- | --- | --- | --- | --- | --- | --- | --- | --- |
|  | **No.** | **%** | **No.** | **%** |  |  | **OR (95%CI)** | **adjOR*** |
| **Hairdresser** |  |  |  |  |  |  |  |  |
| No | 236 | 97.5 | 946 | 97.9 |  |  | Ref | Ref |
| Yes | 6 | 2.48 | 20 | 2.07 |  |  | 1.20 (0.48-3.03) | 1.38 (0.52-3.68) |
|  |  |  |  |  |  |  |  |  |
|  |  |  |  |  |  |  |  |  |
| **Cleaner** |  |  |  |  |  |  |  |  |
| No | 239 | 98.8 | 951 | 234 |  |  | Ref | Ref |
| Yes | 3 | 1.24 | 15 | 3 |  |  | 0.80 (0.23-2.77) | 0.90 (0.24-3.34) |
|  |  |  |  |  |  |  |  |  |
